# Nuclear Export Inhibition Enhances HLH-30/TFEB Activity, Autophagy, and Lifespan

**DOI:** 10.1016/j.celrep.2018.04.063

**Published:** 2018-05-15

**Authors:** Melissa J. Silvestrini, Joseph R. Johnson, Anita V. Kumar, Tara G. Thakurta, Karine Blais, Zachary A. Neill, Sarah W. Marion, Victoria St Amand, Robert A. Reenan, Louis R. Lapierre

**Affiliations:** 1 Department of Molecular Biology, Cell Biology and Biochemistry, Brown University, Providence, RI 02912, USA

## Abstract

Transcriptional modulation of the process of autophagy involves the transcription factor HLH-30/TFEB. In order to systematically determine the regulatory network of HLH-30/TFEB, we performed a genome-wide RNAi screen in *C. elegans* and found that silencing the nuclear export protein XPO-1/XPO1 enhances autophagy by significantly enriching HLH-30 in the nucleus, which is accompanied by proteostatic benefits and improved longevity. Lifespan extension via *xpo-1* silencing requires HLH-30 and autophagy, overlapping mechanistically with several established longevity models. Selective XPO1 inhibitors recapitulated the effect on autophagy and life-span observed by silencing *xpo-1* and protected ALS-afflicted flies from neurodegeneration. XPO1 inhibition in HeLa cells enhanced TFEB nuclear localization, autophagy, and lysosome biogenesis without affecting mTOR activity, revealing a conserved regulatory mechanism for HLH-30/TFEB. Altogether, our study demonstrates that altering the nuclear export of HLH-30/TFEB can regulate autophagy and establishes the rationale of targeting *XPO1* to stimulate autophagy in order to prevent neurodegeneration.

## In Brief

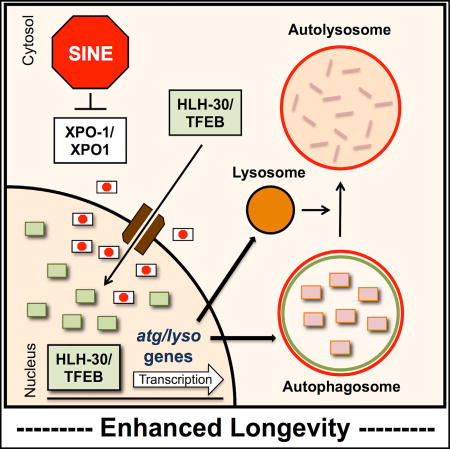

Nuclear enrichment of longevity-associated transcription factors is a conserved feature of multiple long-lived models. Silvestrini et al. show that selective inhibitors of nuclear export (SINEs) increase autophagy via HLH-30/TFEB nuclear enrichment, resulting in longer lifespan in nematodes and ALS-afflicted flies and autophagy/lysosomal enhancements in human cells.

## INTRODUCTION

Autophagy is a conserved cellular mechanism required for longevity across phyla (Lapierre et al., 2015). Macroautophagy (hereinafter referred to as autophagy) consists of the bulk sequestration of intracellular material into a vesicle called the autophagosome, which eventually fuses to the lysosome for degradation. Several different age-related diseases, including neurodegenerative diseases, are characterized by autophagic and lysosomal dysfunctions, which result in the accumulation of unprocessed autophagosomes, aberrant organelles, and aggregates (Nixon, 2013; Wong and Cuervo, 2010). Therefore, to prevent the onset of these age-related diseases, the search for new pharmacological modulators of autophagy is imperative (Galluzzi et al., 2017; Morel et al., 2017). The transcription factor EB (TFEB) preferentially enhances the expression of autophagy-related and lysosomal genes (Sardiello et al., 2009; Settembre et al., 2011) and has emerged as an attractive candidate for autophagy modulation. We and others have uncovered a *C. elegans* ortholog of TFEB called HLH-30 (Lapierre et al., 2013a; O’Rourke and Ruvkun, 2013; Settembre et al., 2013; Visvikis et al., 2014). Functional HLH-30 is required for autophagy induction and life-span extension in multiple longevity models (Lapierre et al., 2013a) and mediates appropriate transcriptional response during starvation (O’Rourke and Ruvkun, 2013; Settembre et al., 2013), as well as heat stress and bacterial infection (Visvikis et al., 2014). Nuclear localization of HLH-30/TFEB is controlled by the conserved regulator, the mechanistic target of rapamycin (mTOR) (Lapierre et al., 2013a). While mTOR inhibitors activate autophagy in part by enhancing the nuclear localization of TFEB (Martina et al., 2012; Roczniak-Ferguson et al., 2012), side effects associated with mTOR inhibition (Kennedy and Lamming, 2016) compel a search for activators of autophagy that are independent of mTOR modulation. Here, we pursued an unbiased approach to find new regulators of the intracellular partitioning of HLH-30/TFEB and uncovered that the conserved gene *Exportin-1 (xpo-1/XPO1)* potently increases the nuclear enrichment of HLH-30/TFEB. Exportins are involved in the nuclear export of proteins, and XPO1 is involved in the recognition and transport of proteins containing leucine-rich nuclear export sequences (Fornerod et al., 1997; Kutay and Güttinger, 2005). In this study, we find that genetic and pharmacological inhibition of XPO-1/XPO1 leads to the nuclear enrichment of HLH-30/TFEB and mTOR-independent autophagic enhancement accompanied by conserved beneficial effects on proteostasis and lifespan.

## RESULTS

### Silencing *xpo-1* Results in Nuclear Enrichment of HLH-30 and Enhanced Autophagy

**Figure 1:**
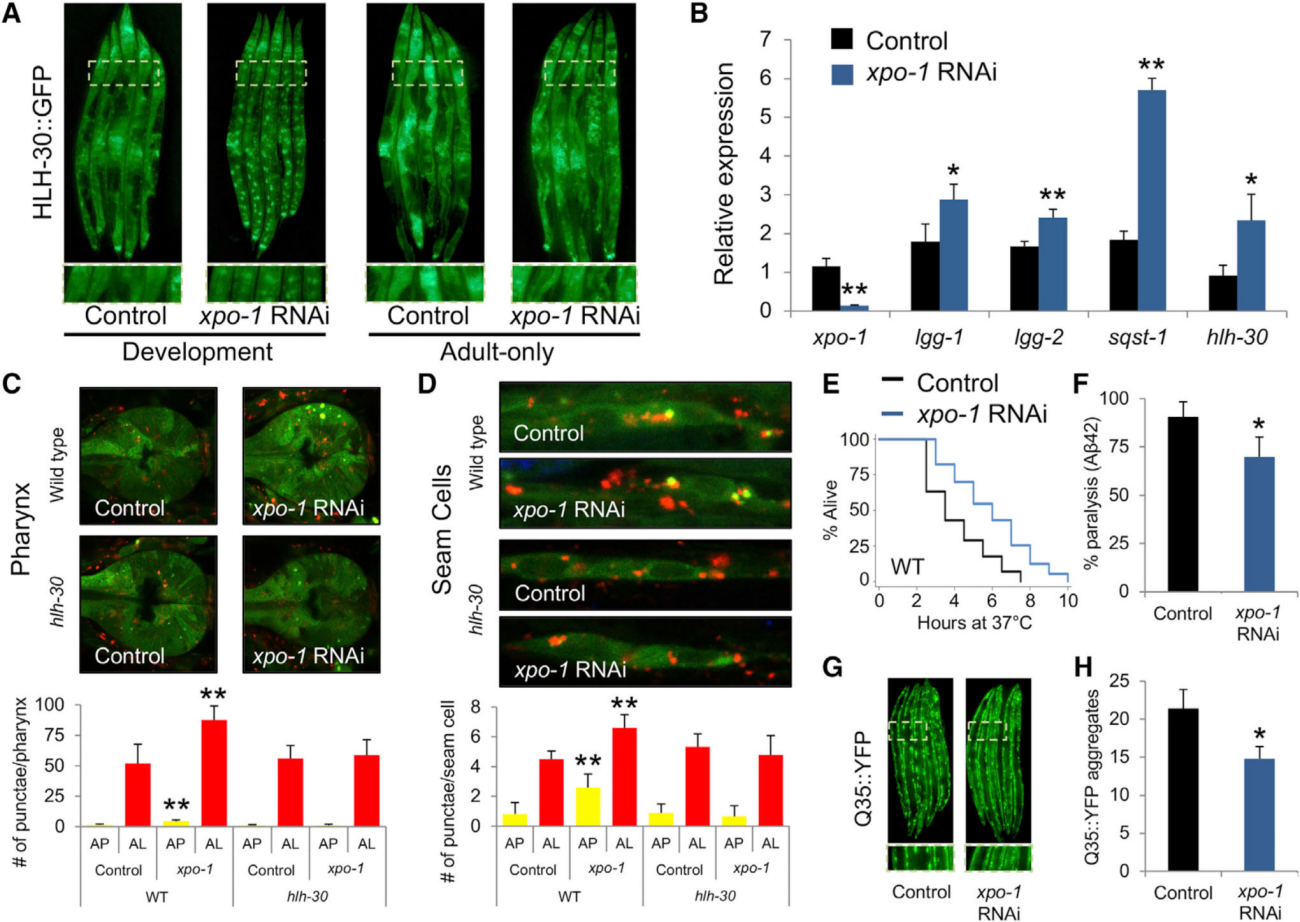
XPO-1 Modulates Nuclear Localization of HLH-30/TFEB and Autophagy (A) Nematodes expressing HLH-30::GFP were fed control bacteria or bacteria expressing RNAi against *xpo-1* during development or during adulthood for 48 hr (1003 magnification). (B) Expression levels of *xpo-1, lgg-1, lgg-2, sqst-1*, and *hlh-30* were measured by qPCR in animals fed control bacteria or bacteria expressing RNAi against *xpo-1* from day 1 to day 5 of adulthood. *p < 0.05; **p < 0.01; n = 4. Error bars represent ± SD, t test. (C and D) Autophagosome (AP) and autolysosome (AL) formation was measured in hypodermal seam cells (C) and pharynx (D) of wild-type and *hlh-30(tm1978)* animals expressing tandem autophagy reporter mCherry::GFP::LGG-1 and fed control bacteria or bacteria expressing RNAi against *xpo-1* for 48 hr during early adulthood. *p < 0.05; **p < 0.01; N = 8. Error bars represent ± SD, t test. (E) Effect of *xpo-1* silencing during 7 days of adulthood on heat resistance was assayed. p < 0.05, n = 200, Mantel-Cox log rank. See Table S1 for statistical analyses and repeats. (F–H) Accumulation of Aβ42 (F) and Q35::YFP punctae (Gand H) were measured in transgenic animals fed control bacteria or bacteria expressing RNAi against *xpo-1* during 5 days of adulthood; images are shown in (G), and quantification is given in (H). *p < 0.05. Q35::YFP, n = 5; Aβ42, n = 100. Error bars represent ± SD, t test.

In order to systematically find new autophagy modulators, we performed a genome-wide RNAi screen and searched for genetic modifiers of the nuclear localization of HLH-30/TFEB by following the distribution of HLH-30 fused to GFP (Lapierre et al., 2013a; Visvikis et al., 2014). Silencing of *xpo-1*, a conserved ortholog of mammalian *Exportin-1* (*XPO1/CRM-1*) (Figure S1), was the most potent inducer of the nuclear localization of HLH-30 (Figure 1A), as enrichment was found in 100% of treated animals, exceeding reported levels in longevity models (Lapierre et al., 2013a; Nakamura et al., 2016). Expression of key autophagy genes and HLH-30/TFEB targets, such as *lgg-1* and *lgg-2 (LC3/GABARAP), sqst-1 (SQSTM1/p62)*, and *hlh-30 (TFEB)* were significantly increased upon *xpo-1* silencing (Figure 1B). To assess autophagy directly, we used a tandem autophagy reporter (mCherry::GFP::LGG-1) that measures autophagosome and autolysosome formation (Chang et al., 2017). Knockdown of *xpo-1* in wild-type animals enhances autophagosome and autolysosome formation in the pharynx and hypodermal seam cells (Figures 1C and 1D). Silencing *xpo-1* in *hlh-30(tm1978)* mutants failed to enhance autophagy (Figures 1C and 1D), demonstrating a direct role for HLH-30 activity in autophagic induction. Animals subjected to *xpo-1* RNAi displayed increased heat resistance, consistent with a role for enhanced autophagy in heat resistance (Kumsta et al., 2017; Visvikis et al., 2014) (Figure 1E and Table S1). Silencing *xpo-1* decreased paralysis in a nematode Alzheimer’smodel expressing Aβ42 (McColl et al., 2012) (Figure 1F) and lowered the formation of Huntington’s–related polyglutamine protein aggregates (Q35::YFP) (Morley et al., 2002; Figures 1G and 1H). Altogether, our data establish a role for the nuclear export protein XPO-1/XPO1 in the modulation of autophagy and proteostasis by regulating the nuclear localization and the activity of HLH-30/TFEB.

### Silencing *xpo-1* Results in Lifespan Extension Requiring HLH-30/TFEB, DAF-16/FOXO, and Autophagy

**Figure 2:**
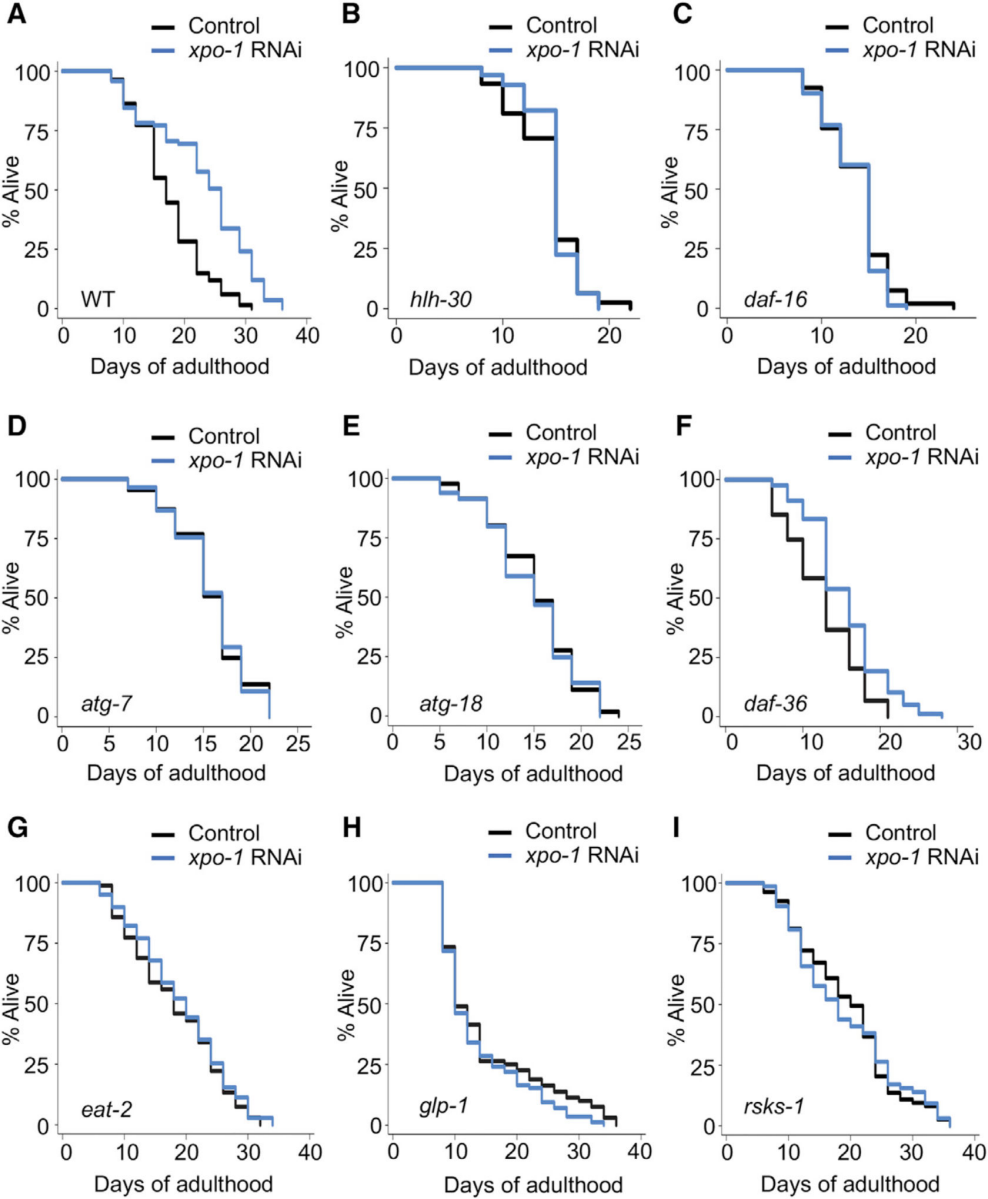
Silencing *xpo-1* Extends Lifespan in *C. elegans* (A–I) Lifespan analyses of (A) wild-type (WT), (B) *hlh-30(tm1978)*, (C) *daf-16(mu86)*, (D) *atg-7(bp411)*, (E) *atg-18(gk378)*, (F) *daf-36(k114)*, (G) *glp-1(e2144),* (H) *eat-2(ad1116)*, and (I) *rsks-1(sv31)* fed control bacteria or bacteria expressing RNAi against *xpo-1* from day 1 of adulthood. n = 100, Mantel-Cox log rank. See also Table S1 for statistical analyses and repeats.

Since elevated autophagy is linked to lifespan extension (Lapierre et al., 2015), we sought to determine how reducing *xpo-1* affected life expectancy in *C. elegans*. Silencing *xpo-1* in adult wild-type animals led to a significant lifespan extension (Figure 2A; Table S1). Interestingly, *xpo-1* displays antagonistic pleiotropy (Kirkwood and Rose, 1991), as whole-life *xpo-1* knockdown decreased lifespan (Figure S2A; Table S1), highlighting developmental roles for XPO-1 followed by a proaging impact in adulthood. Next, we evaluated the contribution of autophagy-related transcription factors and proteins in lifespan extension. Subjecting *hlh-30(tm1978)* or *daf-16(mu86)* mutants to RNAi against *xpo-1* had no effect on their lifespan (Figures 2B and 2C; Table S1). DAF-16 was also found localized in the nucleus of animals subjected to *xpo-1* knockdown, suggesting potential co-regulation with HLH-30 (Figure S2B). Silencing *xpo-1* in autophagy-defective mutants, *atg-18(gk378)* and *atg-7(bp411)*, did not affect lifespan (Figures 2D and 2E; Table S1), demonstrating a requirement for autophagy for the longevity effect associated with *xpo-1* silencing. We found that lifespan extension from *xpo-1* RNAi was not dependent on gonadal signaling using *daf-36(k114)* mutants (Rottiers et al., 2006) (Figure 2F; Table S1). To explore the similarities between *xpo-1* silencing and established long-lived models, we silenced *xpo-1* in dietary-restricted *eat- 2(ad1116)*, germline-less *glp-1(e2144)*, and protein-synthesis *rsks-1(sv31)* mutants. Reducing *xpo-1* expression in these mutants had no additive effect on their lifespan, suggesting mechanistic overlap (Figures 2G–2I; Table S1). Notably, *xpo-1* mRNA levels were low in these three established longevity models (Figure S2C). As observed in several long-lived animals, silencing *xpo-1* resulted in elevated lipid storage (Figure S2D) (Lapierre et al., 2013b; Seah et al., 2016) and increased lysosomal lipase gene expression (Figure S2E) (Lapierre et al., 2011; O’Rourke and Ruvkun, 2013; Seah et al., 2016; Wang et al., 2008). Autophagy gene expression (Figure 1B) was not further enhanced in *glp-1(e2144)* animals subjected to *xpo-1* RNAi (Figure S2F). Taken together, our data indicate that lifespan extension from *xpo-1* silencing mechanistically mimics established longevity models and relies on the activity of HLH-30/TFEB and DAF-16/FOXO, as well as functional autophagy.

### Pharmacological Inhibition of XPO-1 Extends Lifespan

**Figure 3:**
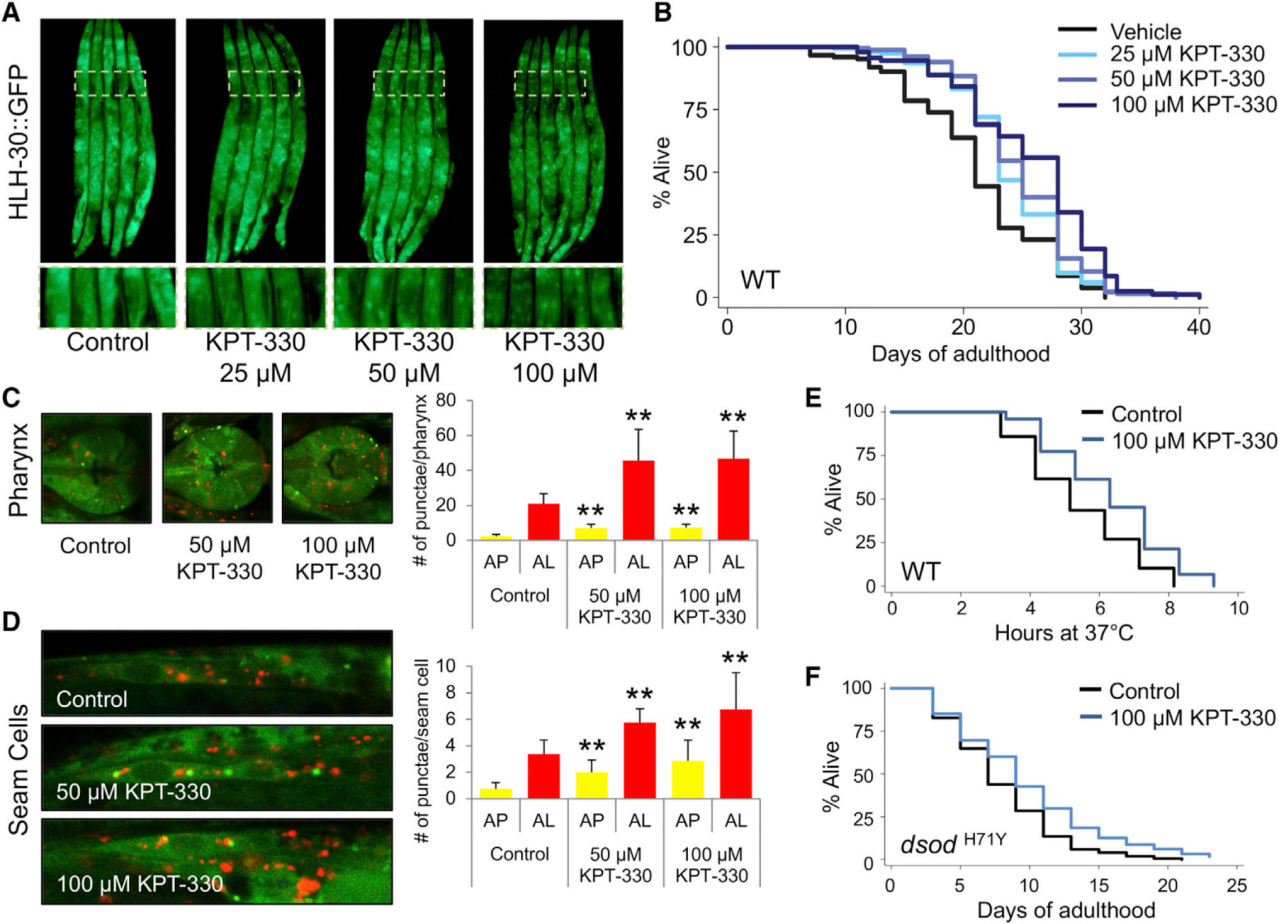
Pharmacological Inhibition of XPO-1/Embargoed Promotes Autophagy and Longevity (A) Day 1 animals expressing HLH-30::GFP were fed *E. coli* OP50 bacteria with DMSO (0.1%) or KPT-330 (25, 50, or 100 µM). 100× magnification. (B) Lifespan analysis of wild-type animals fed bacteria with DMSO (0.1%) or KPT-330 (25, 50, or 100 µM). (C and D) Autophagosome (AP) and autolysosome (AL) were quantified in the pharynx (C) and in hypodermal seam cells (D) of animals expressing mCherry::GFP::LGG-1 and fed bacteria with DMSO (0.1%) or KPT-330 (50 or 100 µM) from day 1 to day 3 of adulthood. **p < 0.01; n = ~10. Error bars represent ± SD, t test. (E) Heat stress assay of animals fed bacteria with DMSO (0.1%) or KPT-330 (100 µM) from day 1 to day 5 of adulthood. p < 0.05; n~100. (F) Lifespan analysis of the ALS model in flies (*dsod^H71Y^*) fed food with DMSO (0.1%) or KPT-330 (100 µM). p < 0.05; n > 300, Mantel-Cox log rank. See Table S2 for statistical analyses and repeats.

Recently developed reversible inhibitors of XPO1 have shown selectivity, bioavailability, and a safe toxicity profile in humans. Among these XPO1 inhibitors, the compound KPT-330 (Selinexor, Karyopharm Therapeutics) can cross the blood-brain barrier, which highlights its potential against neurodegenerative diseases (Green et al., 2015). Thus, we tested the effect of XPO-1/XPO1 inhibition by KPT-330 on autophagy and lifespan in *C. elegans*. Day-1 adults subjected to different concentrations (25, 50, and 100 µM) of KPT-330 for 72 hr displayed nuclear localization of HLH-30::GFP (Figure 3A). These concentrations fell within the typical range used in drug screening studies in *C. elegans* (Benedetti et al., 2008; Ye et al., 2014). We failed to detect nuclear localization of HLH-30::GFP at concentrations below 25 µM (Figure S3A). We observed a significant increase in the lifespan of animals treated with KPT-330 compared to vehicle-treated controls (Figure 3B; Table S2). Similarly, nuclear enrichment of HLH-30 and lifespan extension were observed in animals treated with KPT-276 at 25 µM (Figures S3A and S3B; Table S2). Animals expressing mCherry:: GFP::LGG-1 were treated with vehicle control or KPT-330 (50 or 100 µM) and visualized after 48 hr. Autophagy was enhanced in pharynx and hypodermal seam cells of drug-treated animals (Figures 3C and 3D). Autophagic increases translated into improved resistance to heat (Figure 3E) and a reduction in the formation of polyglutamine-containing (Q40::YFP) aggregates (Mohri-Shiomi and Garsin, 2008) (Figure S3C). Since KPT-330 was able to extend lifespan in nematodes, we sought to analyze its effect on the lifespan of a Sod1-based neurodegenerative model of amyotrophic lateral sclerosis (ALS) in flies (*dsod^H71Y^*) (Sxahin et al., 2017). Feeding KPT-330 (100 µM) to *dsod^H71Y^* flies during adulthood had a beneficial impact on the overall survivorship compared to control (Figure 3F). Specifically, the lifespan of females, but not of males, was extended significantly (Figures S3D and S3E; Table S2), suggesting sex-specific benefits associated with the inhibition of Embargoed/XPO1 and supporting a conserved role for XPO1 in the regulation of proteostasis. Altogether, our data demonstrate that pharmacological inhibition of XPO-1 mimics the effect of *xpo-1* RNAi and protects against neurodegeneration in flies.

### XPO1 Inhibition Leads to TFEB Nuclear Enrichment and Autophagy Enhancement

**Figure 4:**
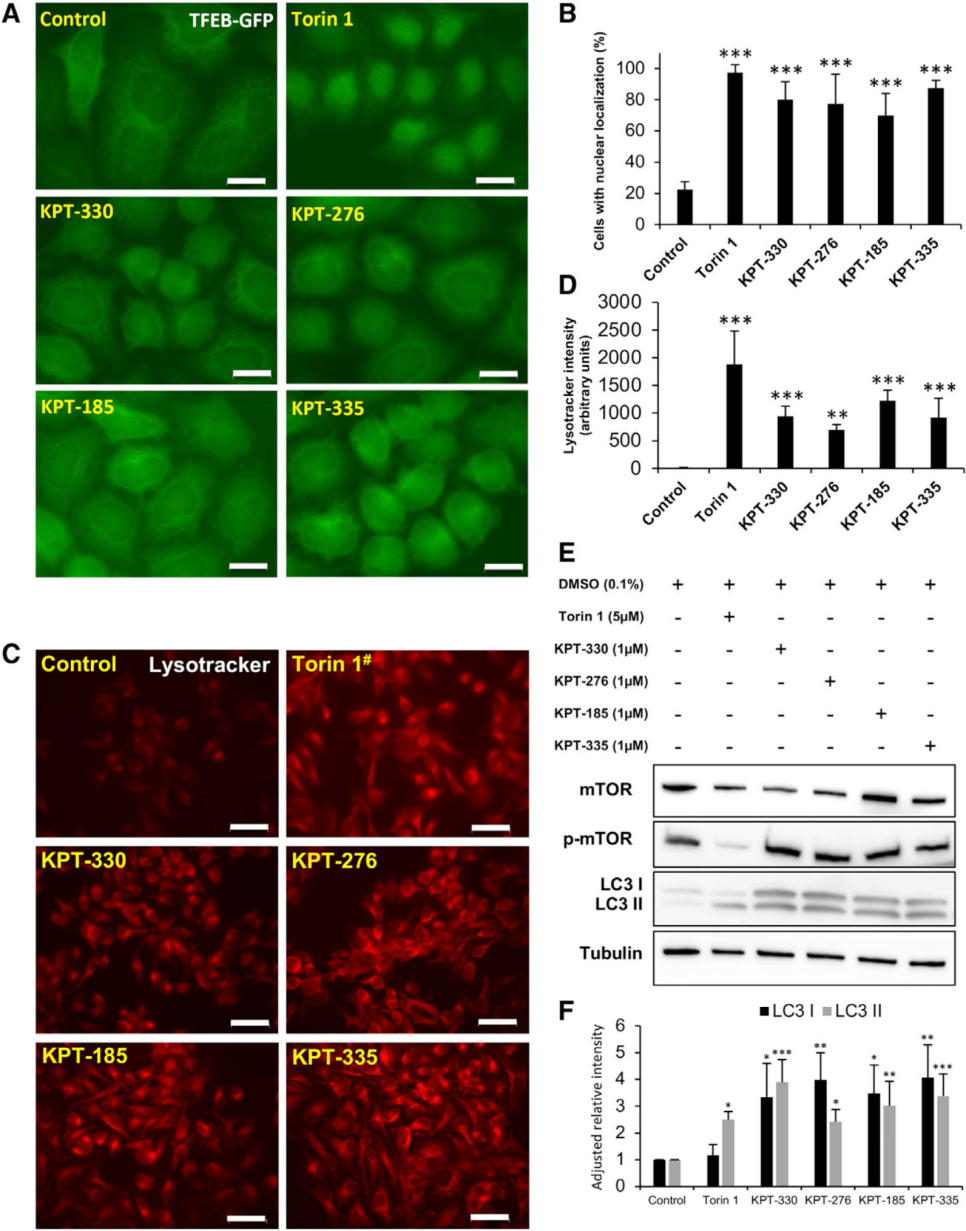
Nuclear Enrichment of TFEB and Autophagy Are Stimulated by XPO1 Inhibition (A) TFEB-GFP-expressing HeLa cells were imaged after incubation for 6 hr in a medium containing vehicle or compounds (Torin 1, 5 µM; KPTs, 1 µM). Scale bars, 20 µm. (B) Percentage of cells with TFEB nuclear localization was quantified from four independent experiments. ***p < 0.001. Error bars represent ± SD, t test. (C and D) HeLa cells were grown in medium containing vehicle or compounds for 6 hr, lysosomes were visualized with Lysotracker Red (C), and signal intensities were quantified (D). **p < 0.01; ***p < 0.001. Scale bars, 50 µm. ^#^Image taken at half the exposure for representational purposes. (E) HeLa cells were grown in a medium containing vehicle or compounds for 24 hr, and proteins were visualized by immunoblotting. (F) Levels of LC3 I and II were quantified by densitometry. *p < 0.05; **p < 0.01; ***p < 0.001. Error bars represent ± SD, one-way ANOVA. Images and blots are representative of three independent experiments.

In order to determine whether the effect of XPO-1/XPO1 inhibition on HLH-30/TFEB and autophagy is conserved in humans, we opted to analyze the effect of XPO1 inhibitors in HeLa cells. Incubating TFEB-GFP-expressing HeLa cells (Roczniak-Ferguson et al., 2012) with various selective inhibitors of nuclear export (KPT-330, KPT-276, KPT-185, and KPT-335) showed a marked increase in nuclear localization of TFEB (Figures 4A and 4B). Similar observations were obtained when cells were incubated with mTOR inhibitor Torin 1, with RNAi against *XPO1*, with GSK-3β inhibitor VIII, or with Leptomycin B (Figures 4A, 4B, S4A, and S4B). Enhanced nuclear localization of TFEB was accompanied by marked increase in red Lysotracker signal, corresponding to an increased presence of acidic lysosomal compartments (Figures 4C and 4D). Enhanced Lysotracker signal from mTOR or XPO1 inhibition required TFEB (Figure S4C). Levels of both forms of LC3 (I and II) were increased under XPO1 inhibition, suggesting an overall upregulation of autophagosome formation and maturation, as seen with LC3-GFP-expressing cells (Lipinski et al., 2010), but the LC3II/LC3I ratio did not significantly differ from that of control (Figures 4E, 4F, and S4D). In contrast to Torin 1, the effect associated with XPO1 inhibition did not rely on reducing mTOR signaling, as phosphorylation of mTOR was not affected by XPO1 inhibition (Figures 4E and S4E). Taken together, these results demonstrate a conserved strategy to enhance autophagy and lysosomal function via TFEB nuclear enrichment by inhibiting the conserved nuclear export protein XPO1.

## DISCUSSION

Our study reveals XPO-1/XPO1 as an evolutionarily conserved modulator of autophagy that regulates the nucleo-cytoplasmic partitioning of HLH-30/TFEB and longevity. Our work highlights the potential for the pharmacological modulation of nuclear export of autophagy-associated transcription factors as an approach for the enhancement of the autophagy process to improve proteostasis and somatic maintenance.

Our unbiased genome-wide RNAi approach identified XPO-1 as a potent regulator of the localization of longevity-associated transcription factors HLH-30/TFEB and DAF-16/FOXO. In addition to providing a strategy to enhance the activity of these transcription factors, our study highlights that their trafficking between cytosolic and nuclear pools is a dynamic process, even in wild-type animals, where these transcription factors are relatively inactive compared to those in long-lived animals (Lapierre et al., 2013a). This is consistent with observations that distribution of HLH-30::GFP (Lapierre et al., 2013a), and also DAF-16::GFP (Berman and Kenyon, 2006), does not display nuclear exclusion. The mechanistic overlap between the lifespan associated with *xpo-1* silencing and longevity models such as the germline-less *glp-1(e2144)*, the dietary-restricted *eat-2(ad1116)*, and the protein synthesis mutant *rsks-1(sv31)* support the hypothesis that nuclear enrichment of autophagy-associated transcription factors is a key converging mechanism in modulating longevity. Longevity models may maintain this partitioning by reducing *xpo-1* expression. Altogether, our study demonstrates that genetic or pharmacological induction of nuclear localization of HLH-30/TFEB in conjunction with DAF-16/FOXO is sufficient to increase autophagy and lifespan.

Several proteins rely on XPO1 for nuclear export (Thakar et al., 2013); as such, our study does not entirely rule out roles outside of HLH-30/TFEB and DAF-16/FOXO in the consequences associated with XPO1 inhibition. For instance, miRNA processing is affected by XPO-1/Embargoed silencing or inhibition (Büssing et al., 2010). In addition, XPO1 inhibition in mammalian cells slows cancer progression by preventing the XPO-1-mediated export of tumor-suppressing proteins (Parikh et al., 2014). Here, we demonstrate that HLH-30/TFEB partitioning is modulated by XPO-1/XPO1, consistent with a report suggesting that TFEB is a likely target of XPO1-mediated nuclear export (Kırlı et al., 2015). Specifically, silencing *xpo-1/XPO1* enhances autophagy, lysosome biogenesis, and longevity via HLH-30/TFEB and without affecting mTOR signaling. Proteostatic and longevity benefits associated with the inhibition of XPO1 are conserved as they extend beyond nematodes and into a Sod-1-based model of ALS in *Drosophila*. Our findings are in line with a study in another ALS fly model (C9ORF72) in which KPT-276 was found to reduce neurodegeneration (Zhang et al., 2015). Together, our findings demonstrate that the use of XPO-1 inhibitors provides an entry point to autophagy and lysosomal regulation by HLH-30/TFEB in an mTOR-independent manner.

Pharmacological modulation of transcription factor function has been relatively challenging (Yan and Higgins, 2013). Here, we present a rationale to alter the nucleo-cytoplasmic partitioning of transcription factors associated with autophagy and longevity via XPO1 inhibition. In particular, this approach may be a promising and viable intervention against diseases associated with proteostatic decline, such as neurodegenerative diseases.

## EXPERIMENTAL PROCEDURES

### Nematode Maintenance

Strains of *C. elegans* were maintained at 20°C on agar plates seeded with *E. coli* OP50 and handled as previously described (Brenner, 1974). See Table S3 for a list of strains used.

### RNAi Screen

Clones from the Ahringer RNAi library (Kamath et al., 2001) were used (Source BioScience). HLH-30::GFP-expressing animals were synchronized by bleaching and put on RNAi-seeded agar plates, incubated at 25°C, and visualized on day 1 of adulthood.

### Fluorescent Microscopy Imaging

Transgenic nematodes were mounted on a 2%agarose pad with 0.1% sodium azide and imaged using a Zeiss Discovery V20 fluorescent microscope.

### Gene Expression Analyses

Nematodes were collected and washed in M9 buffer, and worm pellets were flash frozen with liquid nitrogen. RNA was extracted as described previously (Lapierre et al., 2013a). See Table S4 for details.

### Autophagy Analysis

Nematodes expressing the tandem reporter mCherry::GFP::LGG-1 (Chang et al., 2017) were treated from day 1 of adulthood and imaged using an LSM 800 Zeiss confocal laser scanning microscope, as previously described (Chang et al., 2017).

### Proteostasis Analyses

Heat shock analyses were performed at 37°C, and survival was tracked every hour. Aggregation was visualized by fluorescent microscopy using strains expressing Q35::YFP in muscle (AM140) (Morley et al., 2002) or Q40::YFP in intestine (GF78) (Mohri-Shiomi and Garsin, 2008). Aggregate clearance was assayed with a strain (GMC101) (McColl et al., 2012) inducing expression of human Aβ42 at 25°C. Following a 5-day RNAi feeding, paralysis was assayed after 48 hr at 25°C.

### Lifespan Analyses

Nematodes were synchronized by bleaching, and eggs were fed *E. coli* OP50 during development. Day-1 adults were transferred onto agar plates seeded with RNAi-producing bacteria, and survival was assayed at 20°C. Measurement of lifespan at 25°C of *dsod*^H71Y^ flies was performed as previously described (Şahin et al., 2017).

### HeLa Cell Analyses and Immunoblotting

See the Supplemental Information for details on cell culture conditions, imaging, immunoblotting, and statistical analyses.

## Supplementary Material

1

## Highlights

- Reducing XPO-1/XPO1 increases nuclear HLH-30/TFEB and stimulates autophagy
- Lifespan extension by *xpo-1* silencing mechanistically mimics longevity models
- Selective inhibitors of nuclear export (SINEs) extend lifespan in worms and flies
- SINEs enhance autophagy and lysosomal biogenesis without affecting mTOR signaling
