# Supplementary material for: Nuclear Export Inhibition Enhances HLH-30/TFEB Activity, Autophagy, and Lifespan
Source: Cell Rep. Author manuscript; Available in PMC 2018 Jun 7. (PMC5991088; doi:10.1016/j.celrep.2018.04.063)
Supplement: 1 [file NIHMS970665-supplement-1.pdf]

Cell Reports, Volume *23*

Supplemental Information

Nuclear Export Inhibition Enhances

HLH-30/TFEB Activity, Autophagy, and Lifespan

Melissa J. Silvestrini, Joseph R. Johnson, Anita V. Kumar, Tara G. Thakurta, Karine Blais, Zachary A. Neill, Sarah W. Marion, Victoria St. Amand, Robert A. Reenan, and Louis R. Lapierre

Supplemental Information

Figure S1: XPO-1/XPO1 is a conserved nuclear export protein (Related to Figure 1)

Figure S2: Longevity associated with XPO-1 inhibition mimics longevity models (Related to Figure 2)

Figure S3: Pharmacological inhibition of XPO-1 extends lifespan (Related to Figure 3)

Figure S4: XPO1 inhibition and silencing enhances TFEB nuclear localization and lysosome biogenesis in a TOR-independent manner (Related to Figure 4)

Table S1: Lifespan analyses of animals treated with *xpo-1* RNAi (Related to Figures 1, 2 and S2)

Table S2: Lifespan analyses of animals treated with *xpo-1* inhibitors (Related to Figures 3 and S3)

Table S3: Strains used in this study (Related to Figures 1-3 and S2-S3)

Table S4: Primer list for qPCR (Related to Figures 1 and S2)

Experimental Procedures

Figure S1.

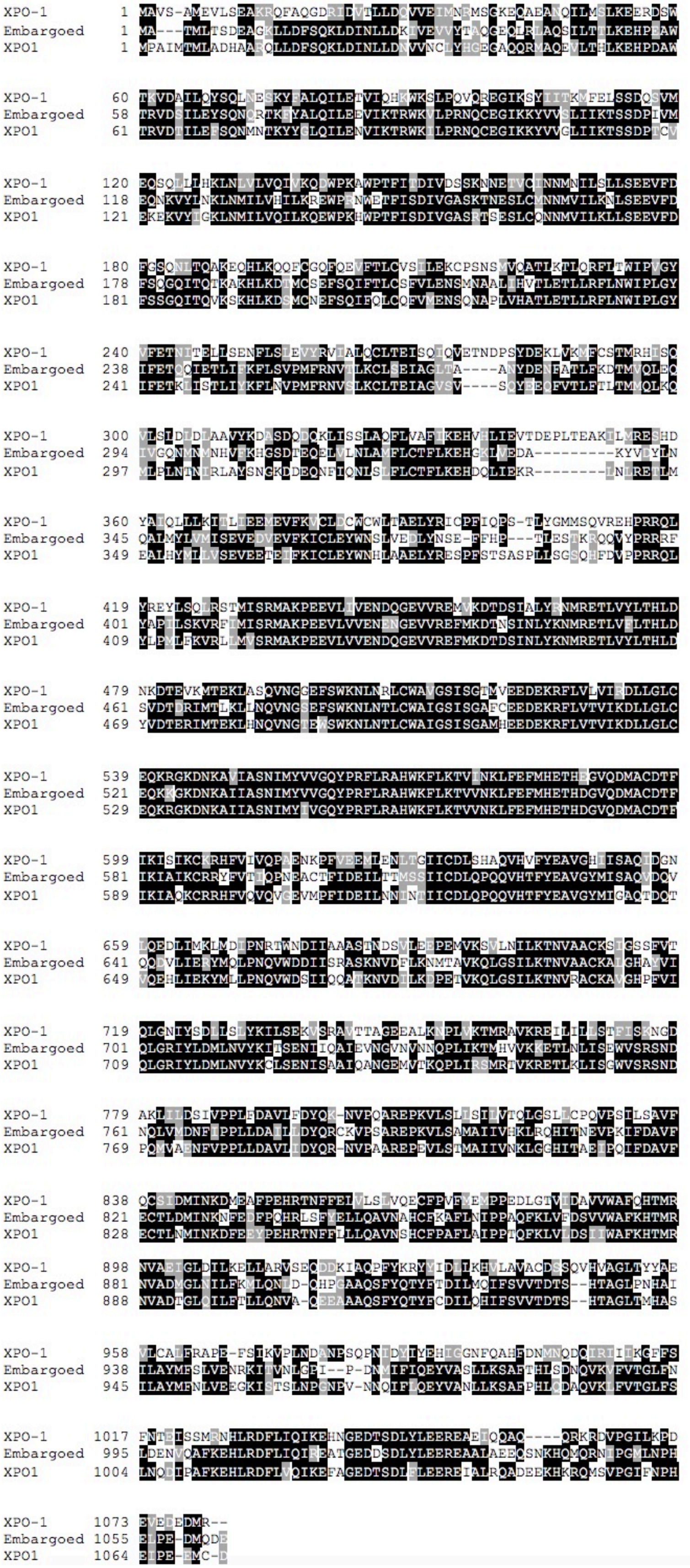

Figure S1. XPO-1/XPO1 is a conserved nuclear export protein (Related to Figure1) Multiple sequence alignment of *Caenorhabditis elegans* (XPO-1), *Drosophila melanogaster* (Embargoed) and *Homo sapiens* (CRM-1/XPO1) performed with T-Coffee and visualized with BoxShade.

Figure S2.

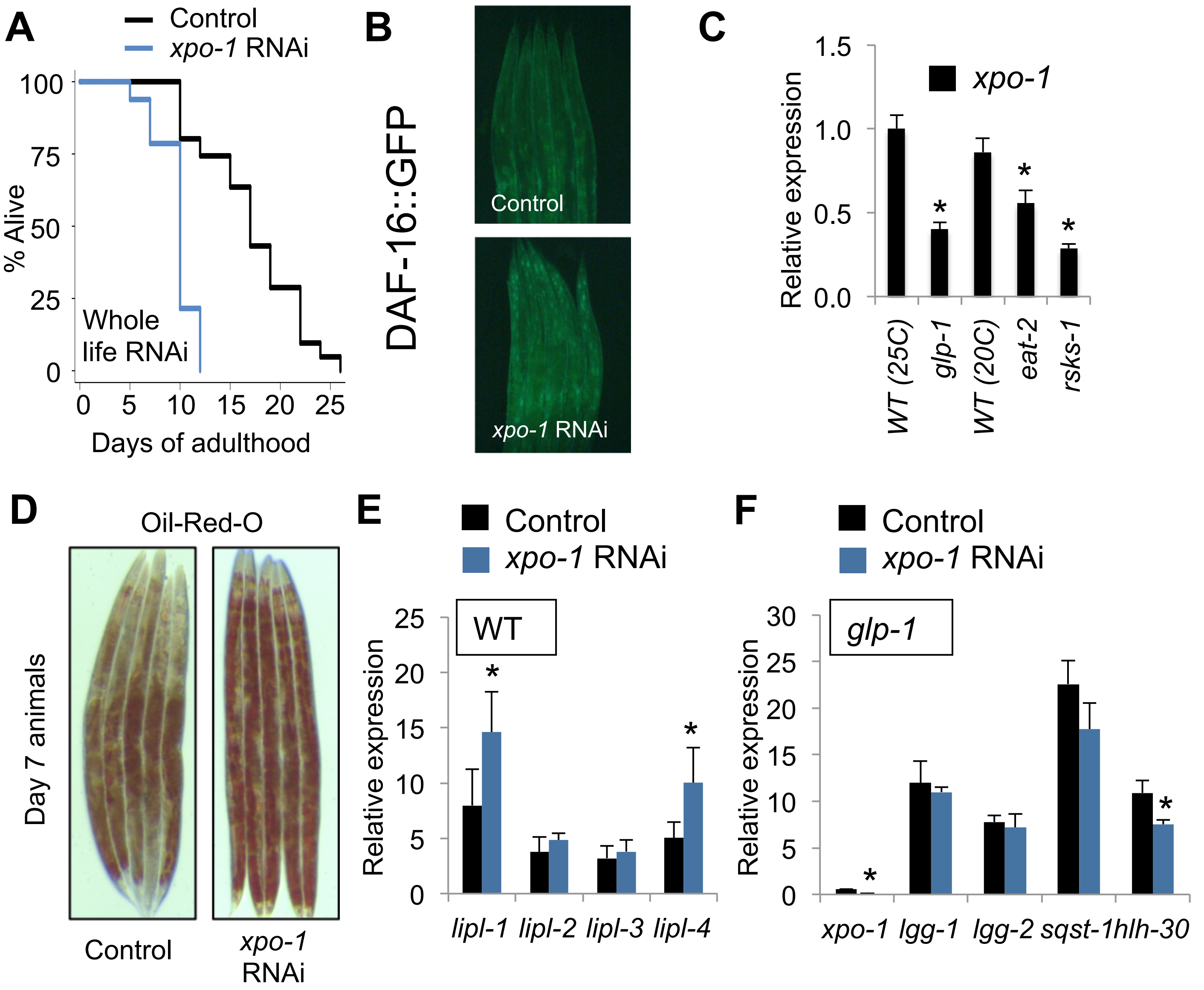

Figure S2. Longevity associated with *xpo-1* inhibition mimics long-lived models (Related to Figure 2) (**A**) Synchronized wild-type eggs were fed throughout lifespan control bacteria or bacteria expressing RNAi against *xpo-1* (See Table S1 for details). (**B**) Day 1 animals expressing DAF-16::GFP (CF1934) and exposed to control RNAi or RNAi against *xpo-1*. (**C**) Levels of *xpo-1* mRNA were measured by qPCR in wild-type (WT) at the non-permissive and permissive temperature (25°C and 20°C, respectively), *glp-1(e2144)* at the non-permissive temperature, *eat-2(ad1116)* and *rsks-1(sv31)*. n=4, *:P<0.05, ±SD *t*-test. (**D**) Oil-Red-O staining of wild-type animals fed for 7 days control bacteria or bacteria expressing *xpo-1* RNAi (**E**) Levels of lysosomal acid lipase genes *lipl-1*, *lipl-2*, *lipl-3* and *lipl-4* in Day 5 wild-type animals fed control bacteria or bacteria expressing RNAi against *xpo-1* since Day 1 of adulthood. n=4, *:P<0.05, ±SD *t*-test. (**F**) Levels of *xpo-1*, *lgg-1*, *lgg-2*, *sqst-1* and *hlh-30* mRNA levels were quantified by qPCR in *glp-1(e2144)* animals fed control bacteria or bacteria expressing *xpo-1* RNAi from Day 1-5 of adulthood. n=4, *:P<0.05, ±SD *t*-test.

Figure S3.

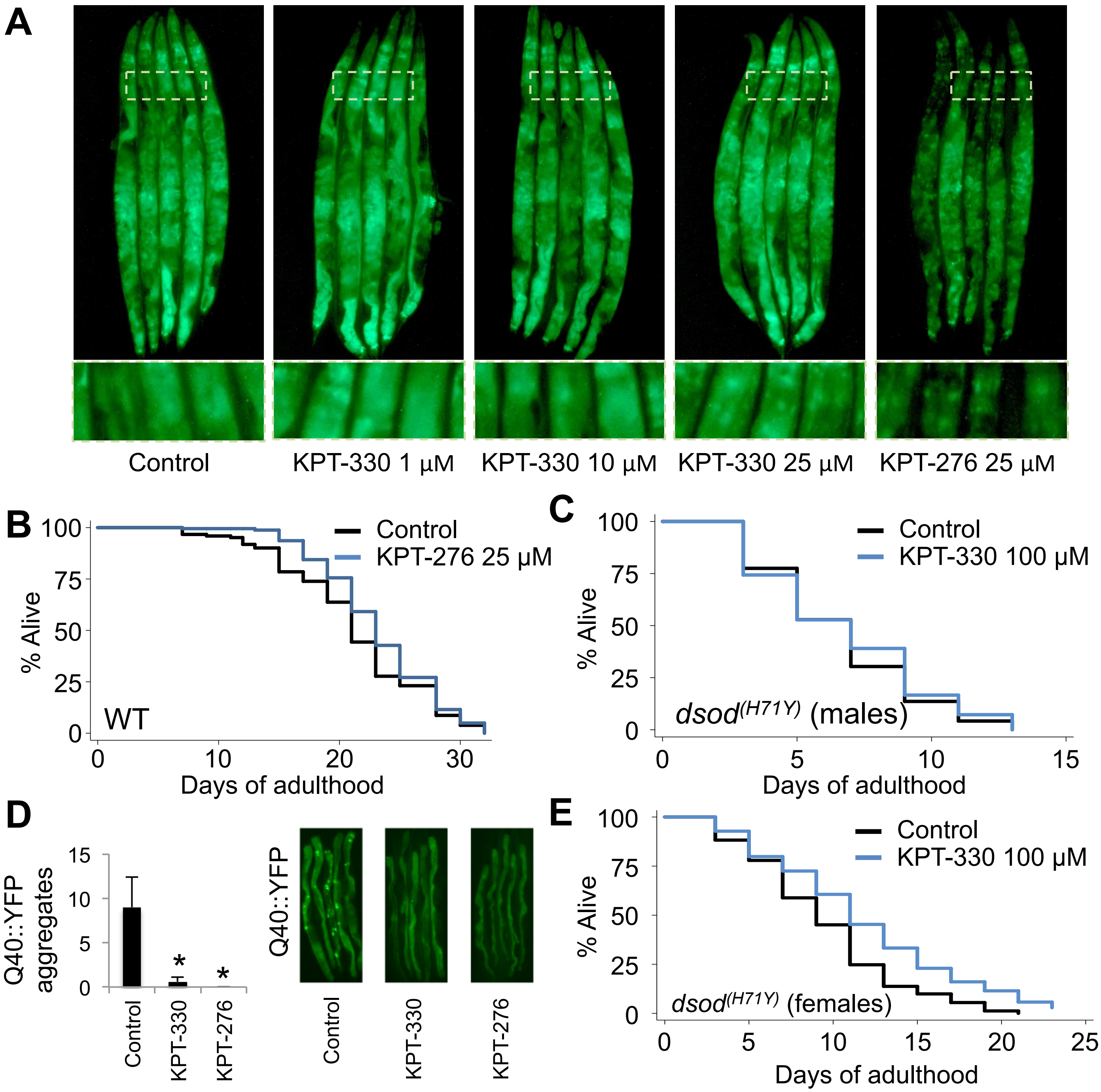

Figure S3. Pharmacological inhibition of *xpo-1* extends lifespan (Related to Figure 3) (**A**) Animals expressing HLH-30::GFP were fed OP50 *E. Coli* bacteria containing vehicle (DMSO 0.1%), KPT-330 (1, 10 and 25 μM) or KPT-276 (25 μM) for 48 hours (100X magnification). (**B**) Lifespan analysis of worms fed OP50 *E. Coli* bacteria containing vehicle (DMSO 0.1%) or KPT-276 (25 μM) during adulthood (see Supplementary Table S2 for details). (**C**) Quantification of Q40::YFP aggregates in day 5 animals fed control OP50 bacteria (DMSO 0.1%) or OP50 bacteria containing 100 μM KPT-330 or 25 μM KPT-276. Micrographs included below histogram (100X magnification) N=5, *:P<0.05, ±SD *t*-test. Lifespan analysis of *dsod*$^{(H71Y)}$ male (**D**) and female (**E**) flies. See Supplementary Table S2 for details.

Figure S4.

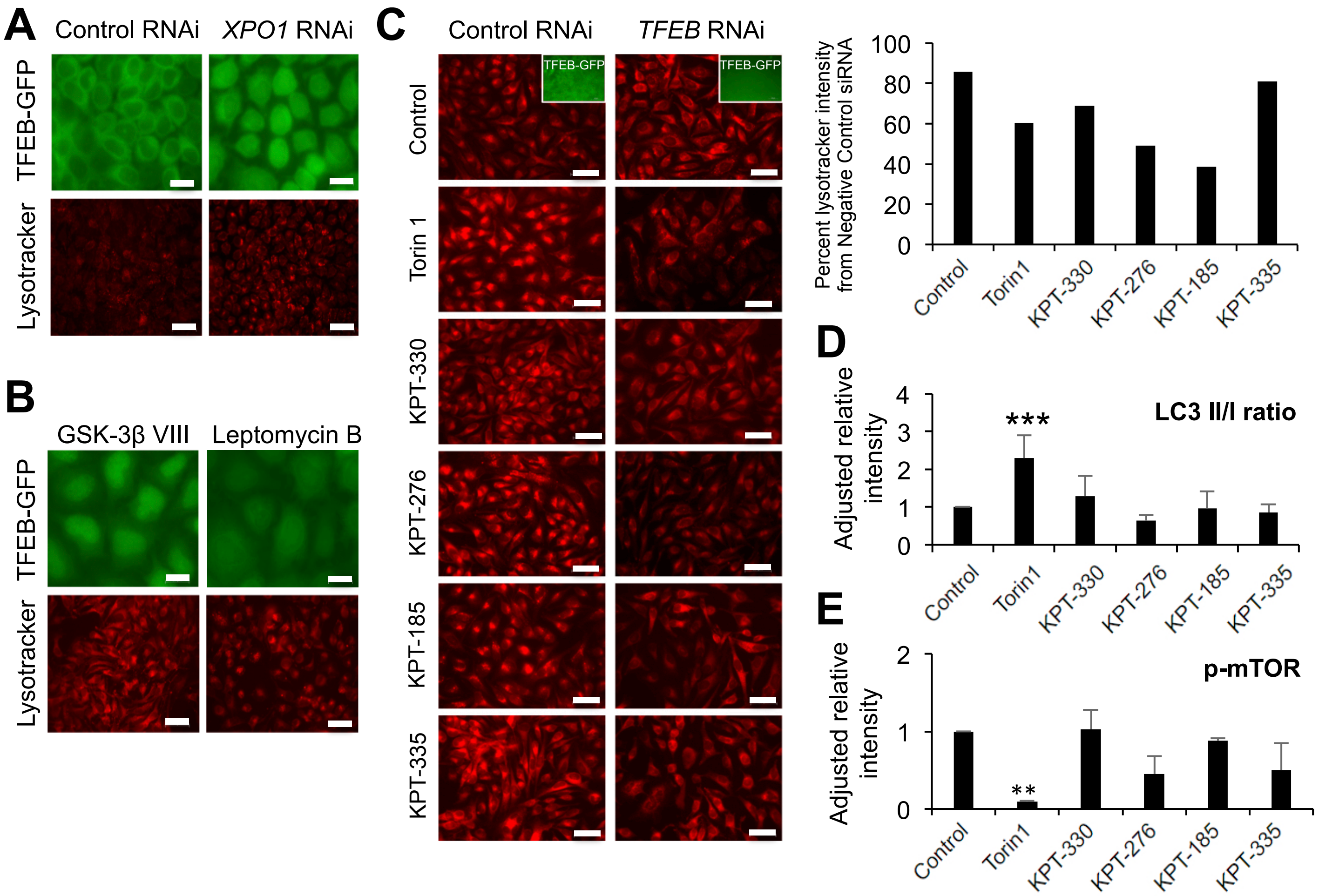

Figure S4. XPO1 inhibition and silencing enhances TFEB nuclear localization and lysosome biogenesis in a TOR-independent manner (Related to Figure 4) (**A**) HeLa cells expressing TFEB-GFP were incubated for 48 hours with Control RNAi or RNAi against *XPO1*. GFP levels (Scale bar = 20 μm) and lysotracker (Scale bar on image = 50 μm) staining were measured. (**B**) HeLa cells expressing TFEB-GFP were incubated for 6 hours with 5 μM of GSK-3β inhibitor VIII or 10 nM of Leptomycin B. (**C**) Hela cells were incubated for 48 hours with Control RNAi or RNAi against *TFEB* and then subjected to 6 hours of DMSO 0.1% (Control), 2 μM of Torin 1 or 1 μM of KPTs (-330, -276, -185, -335). Lysotracker staining was performed (Scale bar = 50 μm). A separate set of HeLa cells expressing TFEB-GFP were used to test the efficiency of TFEB RNAi (see inset). Lysotracker signal was quantified and the % of signal in siTFEB-treated cells vs control is shown. (**D**) Ratio of LC3II/LC3I were quantified from densitometric analyses. ***:P<0.001, ±SD *t*-test (**E**) Densitometric quantification of phospho-mTOR (p-mTOR) immunoblotting shown in Figure 4E and associated independent repeats. Phosphorylated levels of mTOR were normalized with the immunoblots of the corresponding protein. **:P<0.01, ±SD *t*-test

Table S1.

Strains	*xpo-1* RNAi Mean Lifespan (days)	Events Observed	Control RNAi Mean Lifespan (days)	Events Observed	% Difference	P Value
**N2**	20.7	88/100	17.9	69/100	15.6	<0.0001
Wild-type (WT)	23.0	87/100	17.6	68/100	30.7	<0.0001
	19.9	68/100	17.7	49/100	12.4	0.0156
	20.9	88/100	17.9	80/100	16.8	<0.0001
	18.8	48/100	14.4	67/100	30.6	<0.0001
	20.0	65/100	15.5	68/100	28.9	<0.0001
	18.1	69/100	15.0	52/100	20.7	0.0084
	16.7	78/100	14.2	68/100	17.6	0.0138
	22.7	82/100	17.6	77/100	29.0	<0.0001
	24.0	77/100	18.0	52/100	33.3	<0.0001
	22.0	80/100	15.3	56/100	43.3	<0.0001
	21.6	60/100	17.7	61/100	22.0	0.0002
	22.0	66/100	15.7	61/100	40.1	<0.0001
	20.0	81/100	16.4	73/100	21.9	<0.0001
**CF1037**	13.0	89/100	15.0	80/100	-13.3 (15.6)	0.0003
daf-16 (mu86)	13.5	90/100	13.8	91/100	-2.2 (30.7)	0.2635
	12.8	53/100	14.6	58/100	-12.3 (12.4)	0.0004
**LRL1**	15.4	94/100	16.3	71/100	-5.5 (15.6)	0.0040
hlh-30 (tm1978)	14.8	94/100	14.4	82/100	2.8 (30.7)	0.7513
	16.3	78/100	15.6	66/100	4.5 (12.4)	0.3033
**LRL9**	15.2	73/100	15.8	81/100	-3.8 (15.6)	0.9787
atg-7 (bp411)	15.6	96/100	14.2	72/100	9.9 (30.6)	0.0313
	18.1	79/100	16.5	98/100	-8.8 (16.8)	0.0100
**VC893**	14.8	71/100	15.2	73/100	-2.6 (15.6)	0.7583
atg-18 (gk378)	15.4	80/100	16.0	80/100	-3.8 (30.6)	0.4959
	14.7	90/100	14.9	78/100	-1.3 (16.8)	0.9485
**AA292**	16.4	85/100	14.4	82/100	13.9 (15.6)	0.0006
daf-36 (k114)	15.6	78/100	12.7	75/100	22.8 (29.0)	0.0002
	15.2	73/100	13.1	73/100	16.0 (43.8)	0.0292
**CF1903**	14.9	76/100	18.6	70/100	-20.1 (28.9)	0.0011
glp-1 (e2144)	15.6	92/100	17.1	83/100	-8.8 (20.7)	0.2834
	13.7	92/100	14.9	85/100	-8.1 (17.6)	0.1569
**MAH95**	19.9	84/100	19.9	52/100	0.0 (28.9)	0.7297
eat-2 (ad1116)	28.9	74/100	20.7	38/100	-8.7 (20.7)	0.2838
	19.2	75/100	18.3	70/100	4.9 (17.6)	0.4372
**VB633**	18.0	77/100	18.9	68/100	-4.5 (28.9)	0.4634
rsks-1 (sv31)	19.1	70/100	19.5	77/100	-2.1 (17.6)	0.9765
**N2 (WT)**	9.7	76/100	17.2	82/100	-43.6	<0.0001
Whole-life RNAi	10.4	80/100	16.8	73/100	-38.1	<0.0001
**N2 (WT)**	4.9 h.	173/200	3.7 h.	154/200	32.4	<0.0001
Heat Stress	5.9 h.	130/200	4.1 h.	114/200	43.9	<0.0001

Table S1. Lifespan analyses of animals treated with *xpo-1* RNAi (Related to Figures 1, 2 and S2) Details of lifespan of animals fed control bacteria or bacteria expressing RNAi against *xpo-1*. Mean lifespan is displayed in days. Heat stress assays were performed at Day 7 of adulthood (after 7 days of treatment) and survival is reported in hours (h.). Adult lifespan was assayed at 20°C. Change in lifespan between control and *xpo-1* RNAi is displayed as % difference in mean lifespan. Corresponding % difference between control and *xpo-1* RNAi in wild-type animals is reported in brackets. Mantel-Cox log rank statistical analyses were performed using Stata 13.0.

Table S2.

Strains	Treatment	Drug treatment Mean Lifespan (days)	Events Observed	Vehicle control Mean Lifespan (days)	Events Observed	% Difference	P Value
C. elegans							
**N2**	KPT-330						
Wild-type (WT)	25 µM	21.7	93/125	19.3	94/125	12.4	<0.0001
	50 µM	21.2	98/125	19.3	94/125	9.8	<0.0001
	100 µM	22.0	69/125	19.3	94/125	14.0	<0.0001
**N2**	KPT-330						
Wild-type (WT)	25 µM	24.0	136/250	21.2	114/250	13.2	0.0005
	50 µM	24.8	138/250	21.2	114/250	17.0	<0.0001
	100 µM	25.8	84/250	21.2	114/250	21.7	<0.0001
**N2**	KPT-276						
Wild-type (WT)	25 µM	23.2	124/250	21.2	114/250	9.4	0.0293
**N2**	KPT-330 (100 µM)	6.4 h.	75/125	5.4 h.	78/125	18.5	<0.0001
Wild-type (WT)	Heat Stress	6.2 h.	109/125	4.6 h.	90/125	34.7	<0.0001
D. melanogaster							
***dsod*$^{(H71Y)}$**	KPT-330 (100 µM)						
Sod-1 (ALS)	Both sexes	9.8	312/324	7.9	376/387	24.0	<0.0001
	Females	12.0	171/180	9.5	182/187	26.3	<0.0001
	Males	6.8	141/144	6.5	194/200	4.6	0.3116
***dsod*$^{(H71Y)}$**	KPT-330 (100 µM)						
Sod-1 (ALS)	Both sexes	14.1	148/148	10.7	183/183	31.8	0.0005
	Females	18.6	100/100	14.0	141/141	32.9	<0.0001
	Males	5.4	48/48	6.4	52/52	-15.6	0.1047

Table S2. Lifespan analyses of animals treated with *XPO1* inhibitors (Related to Figures 3 and S3) Details of lifespan of animals incubated with OP50-seeded plates with DMSO (0.1%), KPT-330 or KPT-276. Nematode lifespan analyses were carried out at 20°C and fly lifespan analyses were performed at 25°C. Mean lifespan is displayed in days. Heat stress assay were performed at Day 5 of adulthood (after 5 days of treatment) and survival is reported in hours (h.). Variation in lifespan between vehicle control and compound treatment is displayed as % difference in mean lifespan. Mantel-Cox log rank statistical analyses were performed using Stata 13.0.

Table S3.

Strains used in this study	
N2	Wild-type (Kenyon lab - CF)
AA292	*daf-36(k114)* V
AM140	rmIs132 [*unc-54p::Q35::YFP*]
CF1037	*daf-16(mu86)* I
CF1903	*glp-1(e2144ts)* III*
CF1908	*eat-2(ad1116)* II
CF1934	*daf-16(mu86)* I*;* muIs109[*Pdaf-16::gfp::daf-16cDNA* + *Podr-1::rfp*]
GF78	dgEx78 [(pAMS68) *vha-6p::Q40::YFP* + *rol-6*(su1006)]
GMC101	dvIs100 [*unc-54p::A-beta-1-42::unc-54 3'-UTR* + *mtl-2p::GFP*]
LRL1	*hlh-30(tm1978)* IV
LRL9	*atg-7(bp411)* IV
LRL12	*hlh-30(tm1978) IV; sqIs11[lgg-1p::mcherry::gfp::lgg-1* + *rol-6(su1006)]*
MAH215	sqIs11[*lgg-1p::mcherry::gfp::lgg-1* + *rol-6(su1006)*]
MAH240	sqIs17[*hlh-30p::hlh-30::GFP* + *rol-6(su1006)*]
VB633	*rsks-1(sv31) III*
VC893	*atg-18(gk378)* V

Table S3. Strains used in this study (Related to Figures 1-3 and Figures S2-3). *: CF1903 was originally classified as *glp-1(e2141ts)*. Upon sequencing, it was found to instead carry the *e2144ts* allele (see Caenorhabditis Genetics Center website).

Table S4.

Gene	Forward Primer	Reverse Primer	Temp. °C
act-1	CTACGAACTTCCTGACGGACAAG	CCGGCGGACTCCATACC	**60**
cyn-1	GTGTCACCATGGAGTTGTTC	TCCGTAGATTGATTCACCAC	**60**
cdc-42	CTGCTGGACAGGAAGATTACG	CTCGGACATTCTCGAATGAAG	**60**
pmp-3	GTTCCCGTGTTCATCACTCAT	ACACCGTCGAGAAGCTGTAGA	**60**
xpo-1	AAGAACAGGCCGAGGCTAAC	GTTGGACTTGTGGCAACGAC	**60**
lgg-1	ACCCAGACCGTATTCCAGTG	ACGAAGTTGGATGCGTTTTC	**60**
lgg-2	GCATATAACCGTTGCCGAGC	CAAAGCCATCTGGATCACGC	**60**
sqst-1	TGGCTGCTGCATCATCCGCT	TCAATCGTGCCGAGACCGGG	**60**
hlh-30	CTCATCGGCCGGCGCTCATC	AGAACGCGATGCGTGGTGGG	**60**
lipl-1	TGCAACACGGTCTTGAATGC	CCAATCCCAGAATGCCGAGT	**60**
lipl-2	GTTGCTAGCATGTGCCAGTG	TGCCAGAAAGCAGTTTCCGA	**60**
lipl-3	CGATGGGGTTATCCGGCAAT	CGGGCAGGTTCATAGTCCAG	**60**
lipl-4	ACAGGTATTGCGGATGTTTCC	GCATTTGTTCCCCAAATGAA	**60**

Table S4. Primer list for qPCR (Related to Figures 1 and S2).
cDNA were prepared using the iScript Reverse Transcriptase Kit (Bio-Rad). Diluted cDNA of biological quadruplicates were prepared (1/100 dilution) and loaded onto a 96-well plate as technical duplicates. Serial diluted standards (1/25 to 1/400 of pooled cDNA) were included in each 96-well cDNA plates and used to calculate primer efficiency and determine relative levels of mRNA. Diluted samples were loaded on qPCR plates using a Hydra Matrix (Thermo Fisher Scientific). SsoAdvanced Universal SYBR Green Supermix (Bio-Rad) and corresponding primers (above) were added and qPCR plates were run using a Roche 96 Lightcycler. Results from genes of interest were normalized using the geometric mean of 4 housekeeping genes (*act-1*, *cyn-1*, *cdc-42*, *pmp-3*).

Experimental Procedures

Cell Culture

HeLa cells (ATCC) and HeLa cells expressing TFEB-GFP (S. Ferguson lab) were grown in 6-well plates on a coverslip for 24 hours in DMEM High Glucose (Genesee Scientific) containing 2 mM L-Glutamine, 1% Penicillin-Strep and 10% Fetal Bovine Serum (GenClone). For RNAi experiments, both HeLa cell lines were transfected with Silencer Select siRNA against *XPO1* (5 nM) or *TFEB* (10 nM) or negative control siRNA #2 (Thermo Fisher Scientific) with RNAi Max (Thermo Fisher Scientific) and Opti-MEM (Gibco) for 48 hours. Cells were incubated for another 6 hours with DMSO 0.1%, Torin 1 (2 µM), KPT-330, KPT-276, KPT-185 or KPT-335 (1 µM) (Selleckchem). For Lysotracker analysis, cells on coverslips were previously grown with vehicle or compounds [including GSK-3β inhibitor VIII at 5 µM (Millipore Sigma) or Leptomycin b at 10 nM (Millipore Sigma)] for 6 hours followed by a one hour incubation with 100 nM of Lysotracker Red DND-99 (Thermo Fisher Scientific) and live cells were imaged. Coverslips were mounted for imaging onto slides and imaged with a Zeiss Axiovert 200M Fluorescent Microscope. Lysotracker signal was quantified by measuring signal intensity over background of individual cells using ImageJ. For imaging TFEB-GFP signal, cells were fixed in methanol. Nuclear localization was quantified by counting cells that had higher GFP intensity in the nucleus compared to the cytoplasm measured using the software ImageJ.

Immunoblotting

HeLa cells were plated and grown for 24 hours. Thereafter, cells were treated for 24 hours with control or compounds. After 24 hours, cells were collected with a SDS-RIPA lysis buffer (50 mM Tris-HCl, 150 mM NaCl and 1 mM EDTA) including 2% SDS, 1 mM PMSF and Complete ULTRA Protease Inhibitors (Roche). Protein content was measured using the DC Protein Assay (Bio-Rad). 30 µg of proteins were loaded onto 4-15% TGX gels (Bio-Rad) and resolved by electrophoresis. Proteins were transferred using Trans Blot Turbo Transfer System (Bio-Rad) onto nitrocellulose membrane (Bio-Rad). Immunoblotting were conducted using antibodies against Tubulin (ab6160, Abcam), LC3 (ab51520, Abcam), mTOR (2983S, Cell Signaling), phospho-mTOR (5536S, Cell Signaling). Proteins were visualized with ECL reagents (SuperSignal West Pico and West Femto, Pierce) using a ChemiDoc Imaging System (Bio-Rad).

Statistical analyses

Student's *t*-test was used to compare a single parameter between two conditions. One-way Analysis of Variance (ANOVA) was used to compare a single parameter between three or more conditions. In ANOVAs, Dunnett's multiple comparison test was used and all conditions were compared to control. ANOVA was performed using the software GraphPad Prism 7.
